# TESTING THE EFFECTS OF COGNITIVE TRAINING ON ALZHEIMER’S DISEASE AND RELATED DEMENTIAS: THE MODERATING ROLE OF SDOH

**DOI:** 10.1093/geroni/igae098.2187

**Published:** 2024-12-31

**Authors:** George Rebok, Olivio Clay, Gail Wallace, Karlene Ball, Roland Thorpe, Jr., Michael Marsiske, Cynthia Felix, Sherry Willis

**Affiliations:** Johns Hopkins University, Baltimore, Maryland, United States; The University of Alabama at Birmingham, Birmingham, Alabama, United States; Johns Hopkins University, Baltimore, Maryland, United States; University of Alabama at Birmingham, Birmingham, Alabama, United States; Johns Hopkins University, Baltimore, Maryland, United States; University of Florida, Gainesville, Florida, United States; University of Pittsburgh, Pittsburgh, Pennsylvania, United States; University of Washington, Seattle, Washington, United States

## Abstract

The Advanced Cognitive Training for Independent and Vital Elderly (ACTIVE) study is the largest randomized clinical trial of the efficacy of cognitive training (memory, reasoning, processing speed) in improving both cognitive abilities and everyday functioning in normal older adults (N = 2,802; age M = 73.6, range 65-94 years; 76% female; 26% African American), with follow-ups extending through 10 years, and additional follow-ups on claims-based dementia diagnosis through 20 years post-intervention. This presentation considers not only training focus (cognition, exercise) but also a) subjects’ individual differences in social context variables like social determinants of health (SDoH) and b) Alzheimer’s Disease and Related Dementias (ADRD) as training outcome. Overall, we found fewer significant effects of SDoH as moderators of cognitive training effects on diagnosed dementia than we expected, indicating cognitive training was beneficial for individuals across the continuum for most domains of SDoH (e.g., education access, economic stability, neighborhood and built environment, health care access, social and community context). Participants obtained a greater degree of protection from ADRD through cognitive training when they had higher scores for domains associated with health care access (HRs.68 to.75) and more education (HRs.75 to.76). Higher scores on neighborhood and built environment were also associated with lower ADRD risk (HRs.69 to.70). Future work should continue to explore culturally tailored training interventions alone and in combination with physical exercise and environmental modifications such as improved healthcare access to reduce ADRD risk associated with SDoH that disproportionately affects racially diverse aging populations.

